# Development and testing of an evidence-informed structured survey tool for planning tailored technical assistance

**DOI:** 10.1016/j.evalprogplan.2026.102761

**Published:** 2026-01-20

**Authors:** Sarah Moreland-Russell, Jessica Gannon, Kim Prewitt, Louise Farah Saliba

**Affiliations:** a Prevention Research Center, School of Public Health, Washington University in St. Louis, St. Louis, MO 63116, United States; b Center for Public Health Systems Science, School of Public Health, Washington University in St. Louis, St. Louis, MO 63116, United States; c Northwestern Memorial Hospital, Northwestern Medicine, Chicago, IL 60611, United States

**Keywords:** Technical assistance, Evidence-informed, Survey development, Survey tool, Tobacco control programs, Program implementation, Barriers and facilitators

## Abstract

Technical assistance (TA) provides training to improve program development or implementation. This study developed an evidence-informed TA survey tool for planning tailored TA. The survey captures the recipients’ preferred TA delivery methods by activity categories and TA duration and identifies barriers and facilitators to TA engagement. Two phases were carried out: 1) outlining the survey components and assessing face and content validity, and 2) testing the survey with state tobacco control staff and analyzing the survey responses using descriptive statistics. Twenty-seven tobacco control managers and staff representing 14 U.S. state tobacco control programs participated in testing the survey. The final survey included 21 questions. Virtual meetings were the preferred TA delivery method for three TA activities: 1) professional development, 2) coaching and mentoring, and 3) collaborative work. The preferred duration was 1.4–3.0 h/month. Respondents wanted pragmatic information with actionable steps (92 %) and examples of similar challenges other programs address (89 %) in the TA sessions. Respondents pointed to low organizational capacity (76 %) and competing priorities (76 %) as barriers to TA engagement. The TA survey allows TA providers to identify TA recipients’ preferences regarding TA activities and delivery methods, the TA duration, and potential barriers preventing recipients from participating in TA sessions.

## 1. Introduction

Public health programs treat and prevent diseases in the United States by implementing evidence-based interventions (Gottfredson et al., 2015). However, the complexity of implementing these interventions is heightened by variances in the target audience, setting (e. g., school vs. hospital), and context (e.g., urban vs. rural) Wandersman et al., 2012; Chinman et al., 2016). Technical assistance (TA) plays a critical role in mitigating these challenges by providing expertise, resources, and capacity-building support (Wandersman et al., 2012).

The purpose of this study is to describe the development of an evidence-informed structured survey tool to be used in TA planning and delivery. TA is the provision of interactive, individualized education and skill-building to improve the development or implementation of interventions (West et al., 2012). TA encompasses a range of activities, including training, consultation, and implementation support, to ensure that evidence-based interventions are effectively adopted and sustained (Dunst et al., 2019; Durlak & DuPre, 2008). Effective TA can enhance public health outcomes by bridging gaps in knowledge, fostering collaboration, and addressing contextual challenges in diverse settings (Chinman et al., 2016). TA can also be delivered through different modes (Escoffery et al., 2015) and dosages (i.e., frequency, length, and total duration) (Rowbotham et al., 2019). Hence, different public health programs might need distinct components for their TA depending on the specific context of the program. There is still a question of what conditions strengthen a strategy’s focus and how much TA is sufficient (Gayles et al., 2024).

Frameworks and approaches are available in the scientific literature to assist in designing and implementing TA. These approaches range from centralized, expert-driven approaches to more participatory, community-led models (Wandersman et al., 2012). Some TA is delivered through structured frameworks, such as the Active Implementation Framework. This framework designs TA as part of staged and often embedded implementation support. Other frameworks have been designed to be more responsive to immediate needs and tailored for specific audiences. For example, the ‘Getting to Outcomes’ (GTO) framework offers customizable planning, implementation, and evaluation for specific policies and prevention programs, and the interactive systems framework for dissemination and implementation (ISF) emphasizes tailoring TA to delivery systems (Chinman et al., 2004; Wandersman et al., 2000).While understanding of TA structure and implementation approaches has evolved, significant gaps exist in the empirical understanding of how to plan for and deliver TA most effectively (West, 2012; Wandersman & Scheier, 2024). Most frameworks like GTO and ISF tell you *what* to plan and *why*, but they often fall short on the specifics of *how much*, *how often*, and *in what format*. Scott et al. (2022) explain, “advances in the science and practice of TA hinge on understanding what aspects of TA are effective and when, how, and for whom these aspects of TA are effective” (Scott et al., 2022, p.1). Chilenski et al. (2025) note the importance of collaborative working relationships between TA providers and recipients (Chilenski et al., 2025). There is a need for the development of tools to help TA planning and delivery efforts to effectively identify the optimal intensity and duration of TA, delivery format models best suited for the audience to attract attendees and improve their engagement (Metz et al., 2015) and how the delivery format needs to accommodate the context or setting. TA interventions vary in intensity and may include a range of options from one-time training sessions to long-term coaching and embedded support (Rowbotham et al., 2019).

While some tools currently exist to assist in the planning and assessment of TA duration, format, and delivery, they are typically made to uniquely capture specific study needs and TA protocols. For instance, in regard to TA duration, evidence suggests that ongoing TA is more effective in promoting the sustainability of public health interventions than short-term assistance (Fixsen et al., 2009; Metz et al., 2015). Joyce and Showers (2002) also noted that more intensive and sustained TA (e. g., ongoing coaching vs. one-off training) is linked with better outcomes (Joyce & Showers, 2002). However, the field lacks standardized metrics that can be applied across programs to assess how much TA is needed, how frequently it should be delivered, and when diminishing returns occur.

Improving attendance and engagement is critical for ensuring TA effectiveness, yet it is a challenge often reported in public health as funders and implementers recognize that simply offering TA does not guarantee its uptake or effectiveness. Low participation, inconsistent engagement, and lack of follow-through can significantly reduce the impact of TA efforts (Escoffery et al., 2015). Some research has noted the importance of aligning TA content directly to attendee roles, challenges, and organizational context. Wandersman et al. (2012) suggest that TA planning should include a needs assessment and inclusion of partners to increase buy-in and perceived value. Chilenski and colleagues (2016) agreed that a collaborative approach to planning TA has many potential benefits, such as high-quality team functioning and improved TA outcomes (Chilenski et al., 2016). Similarly, Metz et al. (2015) found that recipients were more likely to attend and apply TA when it was linked to their immediate goals, such as achieving funding requirements or addressing prioritized public health issues (Rushovich et al., 2015). As Olson and colleagues note, it is important to tailor TA topics and formats for intended audiences to ensure that the level of training and targeted skills fit the needs of participants (Olson et al., 2021). Studies have also identified solutions to practical barriers like staff turnover and scheduling conflicts that hinder TA attendance. Such solutions include scheduling TA during already established meeting times (Katz & Wandersman, 2016) and frequently reminding attendees with personalized emails or phone calls.

Research also suggests that it is important for TA delivery to be adaptive and flexible, specifically using feedback mechanisms to modify TA as needed. Best practices identified in the literature for TA delivery format include combining different formats (1:1 coaching, group training and coaching, and peer learning) (Fixsen et al., 2009; Joyce & Showers, 2002; Dunst et al., 2019), offering delivery via multiple methods like virtual and in person to increase accessibility (Katz & Wandersman, 2016), and tailoring TA to audience readiness, context, and goals (Wandersman et al., 2012). The COVID-19 pandemic has shaped how we work (Parker et al., 2022) and adding an online or virtual option is important to adapt to telework arrangements (Bennett, 2021).

Public health programs operate in diverse environments with varying levels of organizational readiness, leadership buy-in, and workforce capacity (Durlak & DuPre, 2008; Rushovich et al., 2015), and so TA may need to be tailored to different settings (Escoffery et al., 2015). Unfortunately, there is insufficient research on which contextual factors are most important in shaping the success of TA initiatives. For example, Wandersman et al. (2012) suggest that organizations with strong leadership and pre-existing implementation structures are better positioned to benefit from TA, but the mechanisms through which these factors interact with TA planning and delivery remain underexplored (Wandersman, 2012). Additionally, the mechanisms through which TA contributes to capacity-building and sustainability in public health initiatives are not well understood (Wandersman et al., 2012). Addressing these gaps is crucial to ensuring that TA efforts are evidence-based and capable of maximizing their impact on public health systems.

By developing and validating a structured survey tool for TA planning, this study seeks to enhance the effectiveness of public health interventions through evidence-based and contextually adapted TA. Using best practices for survey development (Artino et al., 2014; Egholm et al., 2020; Rattray & Jones, 2007) we designed this survey tool to help TA planners assess TA methods (what delivery method), TA activities (what format), and TA duration (how much and how often), while also considering engagement barriers and facilitators to maximize TA effectiveness and promote participation. The expectation for this survey tool is to enable a collaborative planning process between TA providers and TA recipients to improve engagement and promote TA effectiveness.

## 2. Methods

### 2.1. Study overview and participants

The development of this TA survey tool was part of a 5-year randomized-control trial study titled Plans, Actions, and Capacity to Sustain Tobacco Control (PACT) (Moreland-Russell et al., 2024; Vitale et al., 2018). We define sustainability as the existence of organizational characteristics that enable a program to implement and continue evidence-based policies and activities over time (Schell et al., 2013). As part of the PACT study, we developed an intervention, the Program Sustainability Action Planning Model and Training Curricula (Moreland-Russell et al., 2024; Vitale et al., 2018), to increase capacity for sustainability and implemented it among state-level tobacco control programs (TCPs) (11 control states and 12 intervention states in the US). The Program Sustainability Action Planning Model and Training Curricula intervention lasted 24 months within each intervention state. It included a 2-day training session with staff and stakeholders. A sustainability action plan was developed, followed by quarterly one-on-one coaching TA with program staff conducted via Zoom to track progress made on the action plan and tailored to address specific challenges in achieving success on the action plan goals. The goal of the Program Sustainability Action Planning Model and Training Curricula was to increase the indicators of program sustainability (i.e., factors that promote the continuation of public health programs when funding is negatively affected or terminated) (Moreland-Russell et al., 2024). When we initiated formative literature to define best practices in TA planning and delivery, we found inconsistency and an overall lack of empirical research in regard to duration, frequency, and format or mode of delivery. Therefore, we added the development of a TA survey tool to the overall research aims to better define, plan, and deliver TA for future use in the facilitation of our training curriculum. The Institutional Review Board of Washington University in St. Louis and the Protocol Review and Monitoring Committee approved the PACT study and TA survey development procedures.

### 2.2. Technical assistance survey development

The development of the TA survey was a two-phase process using a collaborative and iterative approach between the research team and stakeholders. Specifically, we completed the seven steps outlined in Artino et al. (2014) guide to survey design, which includes: (1) conduct a literature review, (2) carry out interviews and/or focus groups, (3) synthesize the literature review and interviews/focus groups, (4) develop items, (5) collect feedback on the items through an expert validation, (6) employ cognitive interviews to ensure that respondents understand the items as intended and (7) conduct pilot testing.

#### 2.2.1. Phase 1: pilot survey creation

The research team identified the potential TA planning survey elements through a formative literature review. The focus of the literature review was centered on TA planning and delivery, including how often, for how long, TA activities, and barriers and facilitators to participating in the TA activities. The literature review also focused on identifying specific TA surveys that included items that assessed TA delivery preferences. During this phase, we also consulted with a TA provider and public health practitioners who regularly received TA. These steps provided direction for drafting our initial survey.

The next step in developing the planning survey involved gathering feedback regarding the face and content validity of the draft survey. Face validity focuses on whether the items of each survey domain are sensible, appropriate, and relevant to the people who use the measure daily. Content validity examines the extent to which the items comprehensively cover the components measured through the survey. We recruited several experts, including one public health consultant, two public health TA providers, and one academic professional, to review the content (face validity). To establish content validity, we obtained written feedback and conducted cognitive response testing interviews with three tobacco control program managers, the intended users of the survey. Tobacco control program managers were asked to complete the survey and answer questions about how they understood items, what they found challenging to answer, and what concepts they thought the items were capturing. After receiving feedback from these groups, we amended the survey tool and created a self-administered online questionnaire using Qualtrics survey software.

#### 2.2.2. Phase 2: survey testing and suitability assessment

We invited 72 tobacco control program staff from the PACT study to test the survey and assess its suitability as a useful collaborative planning tool between TA providers and TA recipients. To ensure an adequate sample size and capture diverse perspectives, we included staff from both intervention and control states in the survey testing. Additionally, we believed that tobacco control program staff from both groups could benefit from the planning tool. Tobacco control program staff are familiar with TA practices and have access to TA offered by the Centers for Disease Control, Office and Smoking and Health, depending on state need. We created and added a hypothetical problem that TA sessions could address in the electronic survey. Due to differences in state tobacco control funding and size, the selected problem was common and intended to be relatable among the survey respondents. We asked participants to focus specifically on TA for the smoke-free policy in their state.

A research team member contacted the state tobacco control program manager through email to explain the purpose of our research and request permission to contact their staff about the survey. A follow-up email was sent to program managers approximately two weeks after our initial email. Once we received approval, we sent emails to TCP staff explaining the purpose of our research and inviting them to participate in the survey. A link to the electronic survey was attached to the email. TCP staff had the opportunity to decline participation in the survey and opt out of future emails regarding the survey. TCP staff were sent two reminder emails after their initial invitation to complete the survey.

To assess the survey’s suitability as a collaborative planning tool between TA providers and recipients, we used descriptive statistics to analyze the survey responses and identify its potential for achieving its purpose. The mean, median, and standard deviation were analyzed using Microsoft Excel version 2402.

## 3. Results

The final survey comprised 21 questions encompassing five categories: (1) technical assistance methods, (2) technical assistance activities, (3) technical assistance duration, (4) technical assistance attendance barriers, and (5) technical assistance engagement facilitators. Questions regarding the respondents’ demographics were also included. The breakdown of the development and testing of the survey is presented below. The final survey can be viewed in Appendix A.

### 3.1. Phase 1: pilot survey creation

Synthesis of the literature review and initial consultation with experts resulted in the initial TA component categories and survey items. Table 1 presents the results of this synthesis. Testing of the face and content validity of the draft survey resulted in the final 21 survey items.

The TA delivery methods category comprised eight survey questions regarding the usefulness of specific types of TA activities and the preferred delivery method for receiving that TA activity. Four different types of TA activities were identified by Dunst and colleagues and included professional development, coaching and mentoring, support and feedback, and collaborative work mediated by a TA provider (Dunst et al., 2019). Feedback from experts during face and content validity testing indicated that it was important that the survey ask about preferred delivery methods for each of the activities. Therefore, we developed four separate survey items to determine the preference for each activity and four corresponding survey questions to determine preference for delivery method for the specific activity. For instance, for professional development we asked “On a scale of 0–10, with 0 being not useful and 10 being extremely useful, please rate professional development as a method for receiving TA to address the issue identified by your program” and, as a follow-up asked, “On a scale of 0–10, with 0 being not wanted and 10 being most wanted, please rate the following delivery methods of professional development for TA” [drop down list of 0–10 for each of the methods]. Feedback from experts also resulted in an expansion of response options for the delivery method item (e.g., real-time webinar, recorded webinar, and virtual platform). We used the scale 0–10 based on research that demonstrates that an 11-point scale when used in usability and perception research can yield more sensitive, discriminative, and reliable results (Finstad, 2010; Nunnally & Bernstein, 1994; Reichheld, 2003).

The TA duration area comprised five survey questions regarding frequency, availability, and desired duration of TA sessions, all of which were informed by survey items developed by Rowbotham et al., (2019). Feedback during face and content validity testing verified the categories used for each of the questions.

Survey items from Escoffery and colleagues, (2015), informed the initial development of the TA engagement barriers and facilitators categories and related survey items. The TA engagement category comprised two survey questions assessing the organizational factors preventing past participation in TA and anticipated organizational factors preventing future participation in TA. The TA engagement facilitators category comprised one survey question assessing elements useful to include in TA. Feedback during face and content validity testing verified the categories used for each of the questions.

### 3.2. Phase 2: survey testing and suitability assessment

For the survey testing and suitability assessment, twenty-seven tobacco control managers and staff participated in piloting the TA survey, representing a response rate of 37.5 %. They answered questions specific to the hypothetical problem that TA sessions could address, specifically, TA for the smoke-free policy in their state. Twenty-five respondents completed the entire survey, while two respondents partially completed it. Respondents represented 14 different state tobacco control programs. The number of respondents from each tobacco control program varied. The regional geographic distribution of respondents as defined by the Centers for Disease Control (Centers for Disease Control and Prevention, 2023) included: Northeast (n = 6, 24.0 %), Midwest (n = 5, 20.0 %), South (n = 8, 32.0 %), and West (n = 6, 24.0 %). Respondents reported holding various professional degrees including an MPH or MHSP (n = 8, 32.0 %); a BS/BA (n = 7, 28.0 %); an MS or MSc (n = 4, 16.0 %); a PhD, DPH, or ScD in a public health field (n = 3, 12.0 %); a MA (n = 2, 8.0 %); a MPA (n = 2, 8.0 %); a RD (n = 1, 4.0 %); and other (n = 1, 4.0 %). Over half of the respondents were managers (n = 13, 52.0 %). An additional 28.0 % (n = 7) identified as directors, while a smaller number of respondents represented coordinators (n = 1, 4.0 %), analysts (n = 1, 4.0 %), and communications (n = 1, 4.0 %). The average time worked in their current position was 2.5 years, while the average time working in public health in any capacity was 15.4 years.

For the TA activities and delivery methods, participants were asked to rate the usefulness of four different types of TA activities to address the hypothetical problem in their state on a scale of 0 (not useful) to 10 (extremely useful). All activities were rated high in regards to usefulness: professional development (M=8.3, SD=1.51), coaching and mentoring (M=8.3, SD=1.88), support and feedback (M=8, SD=2.05), and collaborative work mediated by a TA provider (M=8, SD=1.40). Virtual meetings were the preferred methods of delivery of all the of the TA activities except for support and feedback activities. Table 2 summarizes the preferred delivery methods of each TA activity.

In regard to TA duration, the average optimal frequency for real-time contact for TA sessions participants indicated a minimum of 1.1 sessions per month and a maximum of 1.9 sessions per month. When asked about the optimal frequency for real-time contact TA sessions for their team, the results were similar, with an average minimum of 1.1 sessions and a maximum of 2 sessions per month.

The minimum amount of time respondents said they could allocate to review resources received from TA providers was an average of 1.4 h/month, and the maximum amount of time was an average of 3 h/month. When asked how much time their team could allocate to review resources received from TA providers, the results were slightly higher, with an average minimum of 1.6 h/month and a maximum of 4.7 h/month. Respondents reported the optimal duration of TA was a minimum average of 5.6 months and a maximum average of 11.3 months.

Table 3 summarizes the TA engagement barriers and facilitators. Low organizational capacity and competing priorities were the most cited organizational factors preventing past and future participation in TA. Most respondents chose pragmatic information and actionable steps to enhance TA engagement.

## 4. Discussion

We developed and tested an evidence-informed survey tool to help align the expectations of TA providers and recipients and inform the planning, development, and delivery of TA sessions.

The process of developing the survey involved many systematic steps. Our methodological process was strengthened by gathering evidence from the literature to outline the survey components and later receiving feedback from stakeholders and professional TA providers through an iterative approach. This approach is aligned with recommendations and best practices in survey development (Artino et al., 2014; Egholm et al., 2020; Rattray & Jones, 2007). Our survey supplements existing TA frameworks, such as the Getting To Outcomes (GTO) framework, by efficiently gathering the *how much*, *how often*, and *in what format* “ of planning for TA delivery (Scott et al., 2022; Wandersman et al., 2000). By asking TA recipients about their preferences for frequency, availability, and desired duratiFgon of TA, providers can collaboratively create a TA delivery plan tailored to the end-user’s needs. This collaborative approach to planning TA has many potential benefits, such as high-quality team functioning (Chilenski et al., 2016) and improved outcomes. In addition, our survey addresses considerations for capacity issues by asking TA recipients about both previous and anticipated future factors impacting their ability to participate in TA.

The survey testing and suitability assessment generated a number of interesting results in regard to preferred TA activity, delivery method, and duration. In ranking the usefulness of four different types of TA activities (professional development, coaching and mentoring, support and feedback, and collaborative work mediated by a TA provider), all activities were rated high (8 or above on a scale from 0 to 10). Survey findings indicated a preference for virtual meetings, as they were consistently ranked among the top two preferred delivery methods across all TA activities. Also, for professional development, respondents chose more active learning delivery methods, including real-time webinars or online classes, compared to recorded webinars or interactive websites. The median scores for receiving TA via in-person meetings were below 40 %. These findings are especially relevant in light of how the COVID-19 pandemic has shaped how we work (Parker et al., 2022) and the anticipated continuation of telework options (Bennett, 2021). TA providers need to reassess the preferred delivery methods of TA recipients as our relationship with a virtual work environment continues to evolve, and offering delivery via multiple methods like virtual and in-person will increase accessibility (Katz & Wandersman, 2016). Research also suggests that it is important for TA delivery to be adaptive and flexible, specifically using feedback mechanisms to modify TA as needed (Fixsen et al., 2009; Joyce & Showers, 2002). The selection of the TA delivery format should be guided by careful consideration of the characteristics and complexity of the interventions. TA providers must ensure that training content is suitable for virtual formats and compatible with the level of complexity involved (Olson et al., 2021). When asked about preferred TA delivery methods, no respondents selected “Other,” which suggests that the list of response options provided sufficiently covered the delivery methods of the various TA activities.

In regard to TA duration, participants reported the average optimal frequency for real-time contact for TA sessions as a team and 1 on 1 ranged from 1 to 2 sessions per month. The optimal duration of TA ranged from 5.6 months to 11.3 months. These findings were congruent with evidence that suggests that ongoing TA is more effective than short-term assistance (Fixsen et al., 2009; Metz et al., 2015). Additional research is needed to identify optimal TA duration and intensity.

Pilot results indicate respondents’ preference for pragmatic information and actionable steps to be included in TA sessions. TA recipients also indicated that having examples of how other programs addressed challenges similar to those they are currently facing would be useful. This finding highlights the importance of customizing TA content to meet the specific needs and preferences of the recipients to ensure it can provide tangible guidance and be applied to their unique context (Wandersman et al., 2012; Metz et al., 2015; Olson et al., 2021). As Olson and colleagues note, it is important to tailor TA topics and formats for intended audiences to ensure that the level of training and targeted skills fit the needs of participants (Olson et al., 2021). By understanding these preferences, TA providers can better structure their content to address the needs of TA recipients effectively.

The results also identified potential barriers preventing recipients from fully engaging in and benefiting from available TA. Respondents pointed to low organizational capacity and competing priorities as critical challenges. These challenges align with previous research about the barriers TA recipients face in participating in TA (Durlak & DuPre, 2008; Escoffery et al., 2015; Rushovich et al., 2015). To address these barriers, TA providers can support organizations by sharing strategies to enhance a program’s organizational capacity and manage conflicting priorities.

Although the goal of deploying the survey tool was to test its suitability for informing TA planning, the results of the survey may be used as a starting point by other chronic disease prevention and public health programs when planning TA activities.

Additionally, this survey tool may contribute to the recommendation of the minimum reporting standards for TA evaluation research, as stated by Scott et al. (2022). The tool could support the checklist that the authors provide for describing the TA activities and methods used to measure them.

It is important to acknowledge the limitations of this study. Although we sought input from several stakeholders during the survey’s development, we received fewer responses than expected. Additionally, the response items were not randomized to ensure that the options were not asked in the same order for each respondent. This may have led to a response bias on closed-ended questions. Regarding the limitations in the suitability testing process, the response rate for this pilot may limit the generalizability of the results. However, our sample had a fairly equal geographic distribution of participating state programs. Additionally, this pilot asked participants to focus on a hypothetical scenario that TA sessions could address in their state. The scenario asked participants to consider TA needs for developing smoke-free policy in their state.While this is a scenario that many tobacco control managers face, a real scenario may have prompted respondents to think more specifically about their TA needs, possibly generating different results in regard to their preferences for TA activities or delivery method.

Future research should aim to replicate this study with diverse programs for more generalizable results and could explore TA preferences among other public health programs or topics. Doing so would provide additional insight into the suitability of this survey as a tool for other programs. This would also help better understand TA needs across different program areas, developing more targeted and effective TA approaches. Additional research is also needed to test the utility of this planning survey as a tool in improving outcomes related to TA.

## 5. Conclusion

The development of the TA survey tool was part of a study that aimed to promote the sustainability of state-level tobacco control programs. The survey was developed based on a literature review and feedback from experts and stakeholders. It was tested with staff of the tobacco control programs to determine its suitability as a survey tool for planning TA delivery. This survey offers TA providers a tool to collect data to plan and tailor TA in a collaborative and interactive process with the TA recipients in alignment with research supporting participatory TA (Scott et al., 2024; Ward et al., 2024). The TA survey enables TA providers to understand TA recipients’ preferences concerning TA activities and delivery methods, the length of TA sessions, the facilitators for stakeholders’ engagement in TA, and potential obstacles that might prevent recipients from participating in TA sessions. This survey was developed for tobacco control programs, and future studies may be needed to assess the suitability of the survey with other chronic disease prevention and control and public health programs. By continually refining and improving TA based on recipient needs, we can enhance the impact of TA and better support public health programs to achieve their goals.

### 5.1. Lessons learned

Despite efforts to gather feedback on the survey tool from additional TA providers and TA recipients, we were only able to get written feedback and conduct cognitive response testing with three individuals. While the written feedback and cognitive response testing provided face validity and clarity for the survey questions, additional input and testing would ensure the survey questions make sense to participants and capture the essential information for planning TA. Researchers interested in developing and testing similar tools should consider incentivizing participants to encourage participation.

In addition to the challenges faced in garnering survey feedback, we were challenged by getting survey responses. Including phone calls in our recruitment strategy may have mitigated this challenge. The survey tool’s effectiveness depends on practitioners’ willingness and availability to participate. Therefore, it is also vital that the survey participants are motivated to participate or interested in technical assistance.

## Supplementary Material

1

## Figures and Tables

**Table 1:** Determination of initial TA survey categories and items.

TA planning component category	Survey Item	Source
TA activities	What elements or activities are important to be part of TA sessions? • Professional development [knowledge and skills building] • Technical assistance provider consultation [problem-solving sessions] • Technical assistance support/feedback [input on processes or tasks] • Coaching and mentoring [guiding behavior development and systems change, providing direction, focusing on the big picture]	Question adapted from Dunst et al., 2019
TA methods	How do you prefer to receive a TA? • On-site • Virtual ◦ Real-time webinar ◦ Recorded webinar ◦ Interactive websites • Virtual meeting platform • Calls • Electronic mail Other	Question adapted from Escoffery et al., 2015
TA duration	Realistically speaking, how often are you available for a TA session? • Bi-weekly • Monthly • Quarterly • No preference • Other	Question adapted from Robowtham et al., 2019
TA duration	Realistically speaking, what is the amount of time you can set aside for a TA session? • 30 min • 45 min • 60 min • 90 min • Other • No preference	Question adapted from Robowtham et al., 2019
TA duration	Realistically speaking, for how long would you like to receive TA? • up to 3 months • Up to 6 months • Up to 12 months • Beyond 12 months	Question adapted from Robowtham et al., 2019
TA attendance barriers	What are the barriers to scheduling TA? • Low organizational capacity • Lack of knowledge about the TA providers • Unaware of the topics a TA session can provide • Pressing priorities • Other	Question and options adapted from Escoffery et al., 2015
TA attendance barriers	What are the barriers to attending a TA session? • Program examples too different from my program to be helpful • State travel restrictions not related to cost • Information is typically too basic • No time • Information is impractical for everyday use • Real-world examples are not typically provided • Money to cover travel costs • Other	Question and options adapted from Escoffery et al., 2015
TA engagement facilitators	Which of the following elements would be useful for your program? • Examples of similar challenges being addressed by other programs • Pragmatic information and actionable steps • Additional learning resources • Other	Consultation with experts Options adapted from Escoffery et al., 2015

**Table 2:** Preferred delivery methods by various types of technical assistance activities for Tobacco Control Program staff.

Technical Assistance Activities	Responses *n* (%)
	N =27
Professional Development	
Virtual meeting	23 (85.2)
Real-time webinar	20 (74.1)
Online class	16 (59.3)
In-person workshop	12 (44.4)
Recorded webinar	11 (40.7)
Interactive website	10 (37.0)
In-person class	9 (33.3)
Online videos	9 (33.3)
Email	6 (22.2)
Other	0 (0.0)
No response	0
Coaching and Mentoring	
Virtual meeting	24 (88.9)
Email	15 (55.6)
In-person meeting	10 (37.0)
Phone call	8 (29.6)
Instant messaging	3 (11.1)
Other	0 (0.0)
No response	0
Support and feedback	
Email	22 (84.6)
Virtual meeting	21 (80.8)
Phone call	11 (42.3)
Instant messaging	7 (26.9)
In-person meeting	6 (23.1)
Other	0 (0.0)
No response	1
Collaborative work mediated by a TA provider	
Virtual meeting	22 (84.6)
Presentation	17 (65.4)
In-person meeting	10 (38.5)
Email	7 (26.9)
Instant messaging	5 (19.2)
Phone call	4 (15.4)
Other	0 (0.0)
No response	1

**Table 3:** Barriers and facilitators to engaging in technical assistance reported by Tobacco Control Program staff.

Barriers	Response *n* (%)

	N = 27
Past organizational factors preventing participation in TA	
Low organizational capacity	16 (64.0)
Competing priorities	16 (64.0)
I do not know/I do not remember	3 (12.0)
Not applicable	3 (12.0)
Lack of organizational support	2 (8.0)
Lack of incentives	2 (8.0)
Other	0 (0.0)
No response	2
Anticipated future organizational factors preventing participation in TA	
Low organizational capacity	19 (76.0)
Competing priorities	19 (76.0)
Not applicable	3 (12.0)
Lack of organizational support	2 (8.0)
Lack of incentives	1 (4.0)
Other	0 (0.0)
No response	2
Facilitators	Response *n* (%) N = 27
Useful TA elements for your program	
Pragmatic information and actionable steps	23 (92.0)
Examples that match my program’s problems	22 (88.0)
Additional learning resources	11 (44.0)
Other	0 (0.0)
Not applicable	0 (0.0)
No response	2

## Appendix A. Supporting information

Supplementary data associated with this article can be found in the online version at doi:10.1016/j.evalprogplan.2026.102761.
