# Supplementary material for: Development and testing of an evidence-informed structured survey tool for planning tailored technical assistance
Source: Eval Program Plann. Author manuscript; Available in PMC 2026 Apr 28. (PMC13122650; doi:10.1016/j.evalprogplan.2026.102761)
Supplement: 1 [file NIHMS2163395-supplement-1.docx]

**Appendix A**

**Technical Assistance Survey**

**Introduction**

Thank you for participating in this survey about technical assistance. Your thoughtful completion of this survey will assist us in understanding your preferences for technical assistance implementation as well as potential barriers to receiving technical assistance.

If you have any questions, please feel free to contact us by email.

Thank you.

This survey will ask you questions related to technical assistance (TA). We define TA as the provision of interactive, tailored education and skill-building that involves external expertise and guidance designed to support the effective translation of evidence-based interventions into real-world settings. The term “TA” encompasses a wide range of activities, supports, and resources, such as training, coaching, consulting, modeling, assessing, problem-solving, providing feedback, and assisting with evaluations.

Technical assistance can support your program's implementation of evidence-based interventions (EBIs). Some examples of EBIs to support tobacco prevention and cessation include mass-reach health communications campaigns, increases in the unit price for tobacco products, and comprehensive smoke-free policies. Please answer all questions in this survey with this specific issue - **smoke-free policy** - that would be addressed through technical assistance.

**Technical Assistance Methods**

On a scale of 0 to 10, with **0 being not useful** and **10 being extremely useful**, please rate the usefulness of **professional development** as an activity for receiving TA to support your work on smoke-free policies in your state.

**Professional development:** Encompasses practices to build and strengthen staff, program, organization, and systems capacity. Professional development occurs through activities structured in time and content and may have pre-defined learning outcomes.

**Examples:** classes and training that focus on building knowledge and developing skills for topics related to technical assistance's goals.

0 1 2 3 4 5 6 7 8 9 10

Which of the following delivery methods of **professional development** (i.e., knowledge acquisition and skill-building) TA would you prefer to receive? Select all that apply.

**Professional development:** Encompasses practices to build and strengthen staff, program, organization, and systems capacity. Professional development occurs through activities structured in time and content and may have pre-defined learning outcomes

**Examples:** classes and training that focus on building knowledge and developing skills for topics related to technical assistance's goals.

- In-person workshop
- Real-time webinar
- Recorded webinar
- In-person class
- Online class
- Interactive website
- Virtual meeting (e.g., Zoom, Teams, WebEx platforms)
- Email
- Other ________________

On a scale of 0 to 10, with **0 being not useful** and **10 being extremely useful**, please rate the usefulness of **coaching and mentoring** as an activity for receiving TA to support your work on smoke-free policies in your state.

**Coaching and mentoring:** TA providers use coaching and/or mentoring as part of the provision of TA and in interactions with staff to build and strengthen their organizational capacity.

**Examples:** interactions that guide behavior development to enhance the implementation of the technical assistance's objectives/goals and may include follow-up questions related to the topic.

0 1 2 3 4 5 6 7 8 9 10

Which of the following delivery methods of **coaching and mentoring** would you prefer to receive? Select all that apply.

**Coaching and mentoring:** TA providers use coaching and/or mentoring as part of the provision of TA and in interactions with staff to build and strengthen their organizational capacity.

**Examples:** interactions that guide behavior development to enhance the implementation of the technical assistance's objectives/goals and may include follow-up questions related to the topic.

- Email
- Virtual meeting (e.g., Zoom, Teams, WebEx platforms)
- In-person meeting
- Phone calls
- Instant messaging (e.g., Teams, Slack, etc.)
- Other ________________

On a scale of 0 to 10, with **0 being not useful** and **10 being extremely useful**, please rate the usefulness of **support and feedback** as an activity for receiving TA to support your work on smoke-free policies in your state.

**Support and feedback:** TA providers offer nonjudgmental acknowledgement, encouragement, and feedback on staff efforts toward the accomplishment of changing program practices consistent with the objectives and goals of the plan.

**Examples:** providing input on processes or tasks related to technical assistance's goals and may include follow-up questions related to the topic.

0 1 2 3 4 5 6 7 8 9 10

Which of the following delivery methods of **support and feedback** would you prefer to receive? Select all that apply.

**Support and feedback:** TA providers offer nonjudgmental acknowledgement, encouragement, and feedback on staff efforts toward the accomplishment of changing program practices consistent with the objectives and goals of the plan.

**Examples:** providing input on processes or tasks related to technical assistance's goals and may include follow-up questions related to the topic.

- Email
- Virtual meeting (e.g., Zoom, Teams, WebEx platforms)
- In-person meeting
- Phone call
- Instant messaging (e.g., Teams, Slack, etc.)
- Other ________________

On a scale of 0 to 10, with **0 being not useful** and **10 being extremely useful**, please rate the usefulness of **peer collaborative work mediated by a TA provider** as an activity for receiving TA to support your work on smoke-free policies in your state.

**Peer collaborative work mediated by a TA provider:** Bring together peer organizations so practitioners can learn from others with similar experiences. TA recipients/peers offer collaborative support and feedback on efforts toward performing and accomplishing the objectives/goals of the technical assistance plan; TA providers mediate the process and may provide support and input to TA recipients.

**Examples:** TA recipients exchanging lessons learned through their program practices and sharing approaches to carrying out processes or tasks.

0 1 2 3 4 5 6 7 8 9 10

Which of the following delivery methods of **peer collaborative work mediated by a TA provider** would you prefer to receive? Select all that apply.

**Peer collaborative work mediated by a TA provider:** Bring together peer organizations so practitioners can learn from others with similar experiences. TA recipients/peers offer collaborative support and feedback on efforts toward performing and accomplishing the objectives/goals of the technical assistance plan; TA providers mediate the process and may provide support and input to TA recipients.

**Examples:** TA recipients exchanging lessons learned through their program practices and sharing approaches to carrying out processes or tasks.

- Email
- Virtual meeting (e.g., Zoom, Teams, WebEx platforms)
- In-person meeting
- Phone call
- Instant messaging (e.g., Teams, Slack, etc.)
- Presentation sharing lessons learned from common programs/projects
- Other ________________

**Technical Assistance Duration**

Considering your project, staffing, and other priorities, please answer the following questions about the **frequency, availability, and desired duration** of TA that would be most useful to support your work on smoke-free policies in your state.

What would be the optimal frequency for **real-time contact** TA sessions **for you**?

Minimum number of **session(s)** per month: _____

Maximum number of **session(s)** per month: _____

What would be the optimal frequency for **real-time contact** TA sessions **for your team**?

Minimum number of **session(s)** per month: _____

Maximum number of **session(s)** per month: _____

How much time could **you** allocate per month to **review resources** received from TA providers? Please use decimals for fractions of an hour (e.g., 1.5 for one and a half hours).

Minimum number of **hours** per month: _____

Maximum number of **hours** per month: _____

How much time could **your team** allocate per month to **review resources** received from TA providers? Please use decimals for fractions of an hour (e.g.,1.5 for one and a half hours).

Minimum number of **hours** per month: _____

Maximum number of **hours** per month: _____

What would be the **optimal duration** of TA to support your work on smoke-free policies in your state?

Minimum number of months: _____

Maximum number of months: _____

**Technical Assistance Attendance**

Which of the following **organizational factors** have **prevented** you from participating in TA in the **past**? Select all that apply.

- Low organizational capacity (e.g., staff turnover)
- Lack of organizational support (e.g., importance to leadership; leadership values and expectations; commitment to excellence; a culture that supports innovation)
- Lack of incentives (e.g., professional recognition; appeal to altruism/greater social good; monetary gain)
- Competing priorities (e.g., staff reallocation to COVID-19; new Notice of Award)
- Other ________________
- Not applicable
- I do not know / I do not remember

Which of the following **organizational factors** do you **anticipate preventing** you from participating in TA in the near **future**? Select all that apply.

- Low organizational capacity (e.g., staff turnover)
- Lack of organizational support (e.g., importance to leadership; leadership values and expectations; commitment to excellence; a culture that supports innovation)
- Lack of incentives (e.g., professional recognition; appeal to altruism/greater social good; monetary gain)
- Competing priorities (e.g., staff reallocation to COVID-19; new Notice of Award)
- Other _______________
- Not applicable

**Technical Assistance Engagement**

Which of the following **TA elements** would be useful for your program? Select all that apply.

- Examples of similar challenges being addressed by other programs
- Pragmatic information and actionable steps
- Additional learning resources
- Other _______________
- Not applicable

**Other Information**

Please select your professional degree(s). Select all that apply.

- Associate degree
- BS/BA
- BSN
- LPN
- MA
- MD/DO
- MPA
- MPH or MSHP
- MS or MSc
- PhD, DPH, or ScD in a public health field
- PhD or ScD in another field
- RD
- RN
- Other ________________
- Not applicable

What is your current position in the organization?

What state does your organization serve?

How long have you worked in your current position?

_______ year(s)

How long have you worked in public health in any capacity?

_______ year(s)
